# Tailoring Exergames for Clinical Populations: Lessons Learned and Design Considerations for Persons With Dementia and Late-Life Depression

**DOI:** 10.7759/cureus.83502

**Published:** 2025-05-05

**Authors:** Sarah C Pistritto, Andrew Hogue, Sara Elgazzar, Pritika Lally, Winnie Sun

**Affiliations:** 1 Faculty of Health Sciences, Ontario Tech University, Oshawa, CAN; 2 Faculty of Business and Information Technology - Game Development and Interactive Media, Ontario Tech University, Oshawa, CAN; 3 Faculty of Science, Ontario Tech University, Oshawa, CAN

**Keywords:** cognitive stimulation, exergaming, late-life depression, persons with dementia, zumba

## Abstract

Exergames have emerged as promising tools for enhancing physical and cognitive engagement among various populations. However, standard commercial exergames often follow a “one-size-fits-all” approach, which may not be effective in addressing the unique needs of clinical populations such as individuals with dementia and late-life depression (LLD). This paper explores the challenges and insights gained from tailoring exergames specifically for these groups. By detailing the design and development process, we highlight key lessons learned, including the importance of customizing game mechanics, user interfaces, and feedback systems to align with the cognitive and physical capabilities of these individuals. This paper further proposes design considerations and best practices for future research, aiming to maximize therapeutic outcomes, user engagement, and overall feasibility of exergame interventions.

## Introduction

Exergames (exercise + gaming) are a novel technological approach to promote the engagement and uptake of physical activity (PA) and various cognitive activities. Exergames can increase motivation and were found to improve an individual’s function, balance, and mobility [1,2]. However, commercial exergames encompass a “one-size-fits-all” approach that does not consider the specific needs and preferences of the end user to maximize their therapeutic targets, engagement, and needs [3]. In particular, dementia and late-life depression (LLD) present unique challenges that require tailored interventions to support their cognitive and physical well-being. Standard exergames are designed with a broad audience in mind, not considering the requirements of these clinical groups. A challenge for the development of exergames is knowing the design considerations for each population and how to integrate different components to maximize user uptake [3]. Research has highlighted the potential of PA as a therapeutic intervention for both LLD and dementia to alleviate symptoms of depression and low mood and enhance cognitive function [4-6]. However, many older adults face barriers to engaging in traditional forms of exercise due to mobility issues, lack of motivation, or cognitive impairments that make structured exercises challenging [7-9]. Visual feedback, rewards, and progression can motivate users to stay active and engaged, while customizing exercises and cognitive activities can enhance motor skills and function [10]. The main goal of this paper is to share lessons learned from designing an exergame for persons with dementia and LLD and propose design considerations for future researchers to create effective interventions.

## Materials and methods

Sample characteristics 

This mixed methods cohort feasibility study included five participants (one male and four female) diagnosed with LLD (as confirmed by medical diagnosis) or dementia (as confirmed by medical diagnosis and assessed using the Mini-Mental State Exam (MMSE)). All participants (n = 5) were screened and included in the study.

The MMSE scale was used as a pre-screening tool to identify individuals with dementia for participation in the study. Participants with MMSE scores ranging from 12 to 24, indicating mild to moderate dementia, were included. Additional criteria included participants aged 60 and older and receiving services from the Alzheimer’s Society of Durham Region or Ontario Shores Centre for Mental Health Sciences (in-patient or outpatient). Exclusion criteria included a history of auditory or visual impairments, dry mouth or eyes, oral lesions, autoimmune diseases (affecting saliva collection), electroconvulsive therapy (ECT), difficulty communicating in English, and MMSE scores below 12 or above 25. Study exergaming sessions were conducted at Ontario Tech University and Ontario Shores Centre for Mental Health Sciences. Ethical approval was obtained from the Research Ethics Boards of Ontario Shores (JREB #22-033-B) and Ontario Tech University (REB # 17059).

Table 1 presents the characteristics of the participants.

**Table 1:** Participant characteristics ASDR: Alzhiemer's Society of Durham Region.

Participant	MMSE score	Gender	Site	Diagnosis
P-11	24	M	ASDR	Cognitive impairment
P-34	29	F	Outpatient	Late-life depression
P-51	24	F	Outpatient	Cognitive impairment
P-73	24	F	Outpatient	Cognitive impairment
P-81	26	F	Outpatient	Late-life depression

Exergame intervention

A pilot cohort feasibility intervention involved an approximate 30-minute exergame, with PA Zumba and cognitive stimulation (CS) activities completed twice a week for four weeks (eight times in total). The intervention dosage was based on previous therapeutic aerobic exercise interventions with the same frequency or dosage [9-11], which implemented similar protocols with sessions conducted 2-3 times per week over a period of 4-6 weeks. Consistent with these studies, this study followed a structured aerobic exercise design. In this intervention, participants alternated between a PA Zumba song and a CS activity. Each session consisted of a total of five PA Zumba songs and four CS activities. Afterward, the participants participated in a semi-structured one-on-one interview to explore the impact on their mental health and well-being, as well as identify facilitators and barriers.

Exergame design

**Figure 1:**
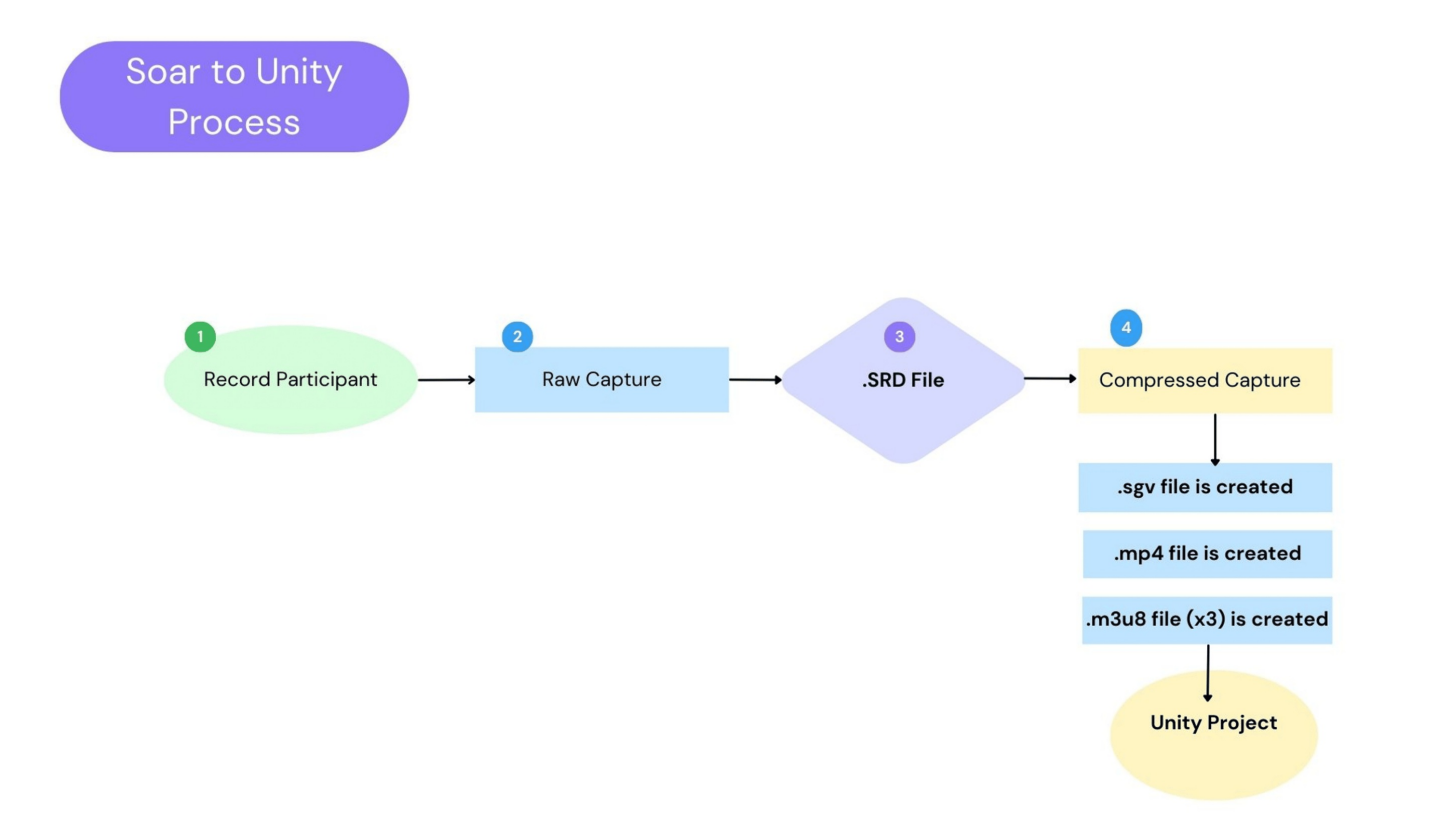
Steps for SOAR to Unity processing SOAR: Security Orchestration, Automation, and Response. Image Credit: Authors' original creation.

This exergame uses a Microsoft Azure Kinect DK model number 1880 camera (manufactured in China in 2019) to track participants' movements through 3D volumetric models. Unity Software (Unity Technologies, San Francisco, CA, United States) works with the Azure Kinect to create the exergame, detecting 1-2 people at a time. The Security Orchestration, Automation, and Response (SOAR) volumetric capture system was used to create the 3D models, with five models recorded using seven Record Depth (RGBD) Azure Kinect cameras to enhance video quality. Cameras were positioned 1-2 m from the center and calibrated with the provided cube (SOAR) before recording. Lighting was controlled in the lab, and blinds were closed to reduce infrared light. Zumba songs were choreographed and recorded by two volunteers and the game designer. Raw recording files were captured and compressed in SOAR to create a Unity-compatible SRD file (Figure 1). 

Menu design

**Figure 2:**
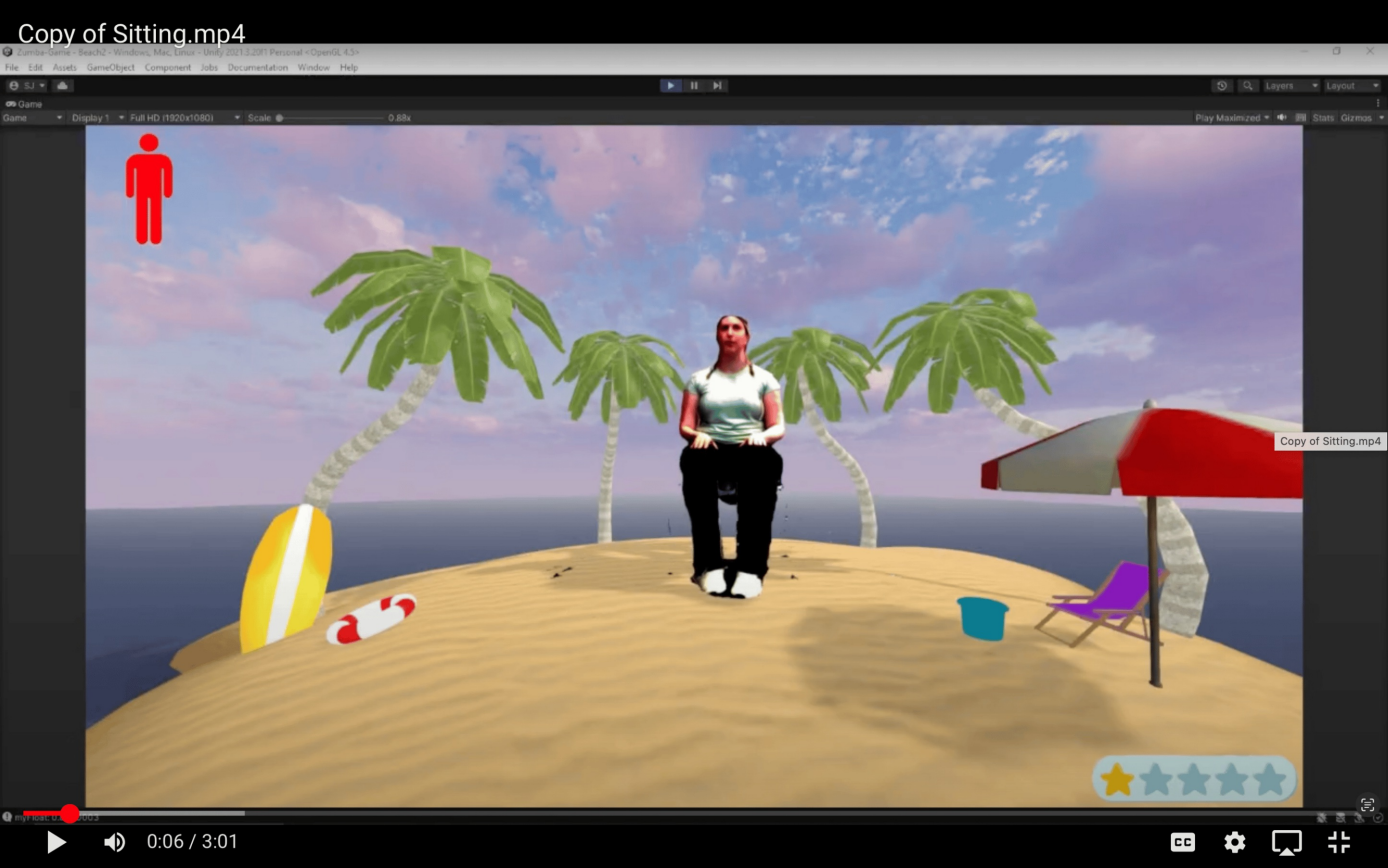
Photo of Zumba avatar Image Credit: Authors' original creation.

Users have the option to select from three background choices (beach, park, and snow mountains) that were created in Unity with the terrain tool (Figure 2). Skybox was used to enhance the background lighting. Two PA modes were created: (1) standing and (2) seated. Participants can pick which Zumba version they would like to follow. A seated option was created for those with physical impairments who can still participate in the activities. Once selected, a play button appears, and participants can begin the exergame. 

Physical activity design

Zumba was chosen as the mode of PA because there is no “correct” way to perform it. Given its emphasis on repetition and movements to the beat, this allows individuals to move within their own boundaries creating a safe space [12-14]. This reduces fall risk, allowing participants to move freely within their own boundaries. Additionally, Zumba has not been pilot-tested in this demographic to assess its feasibility as a form of PA. The exergame includes five Zumba dances of varying durations to follow an aerobic exercise plan. It starts with a warm-up, followed by two moderate-intensity songs, reaching peach exertion before cooling down. Each song was choreographed to encourage continuous movement, while allowing for rest periods during transitions to CS activities. This aerobic plan aims to boost engagement and motivation for better health outcomes. All songs used were copyright-free [15-19].

Cognitive stimulation activities

**Figure 3:**
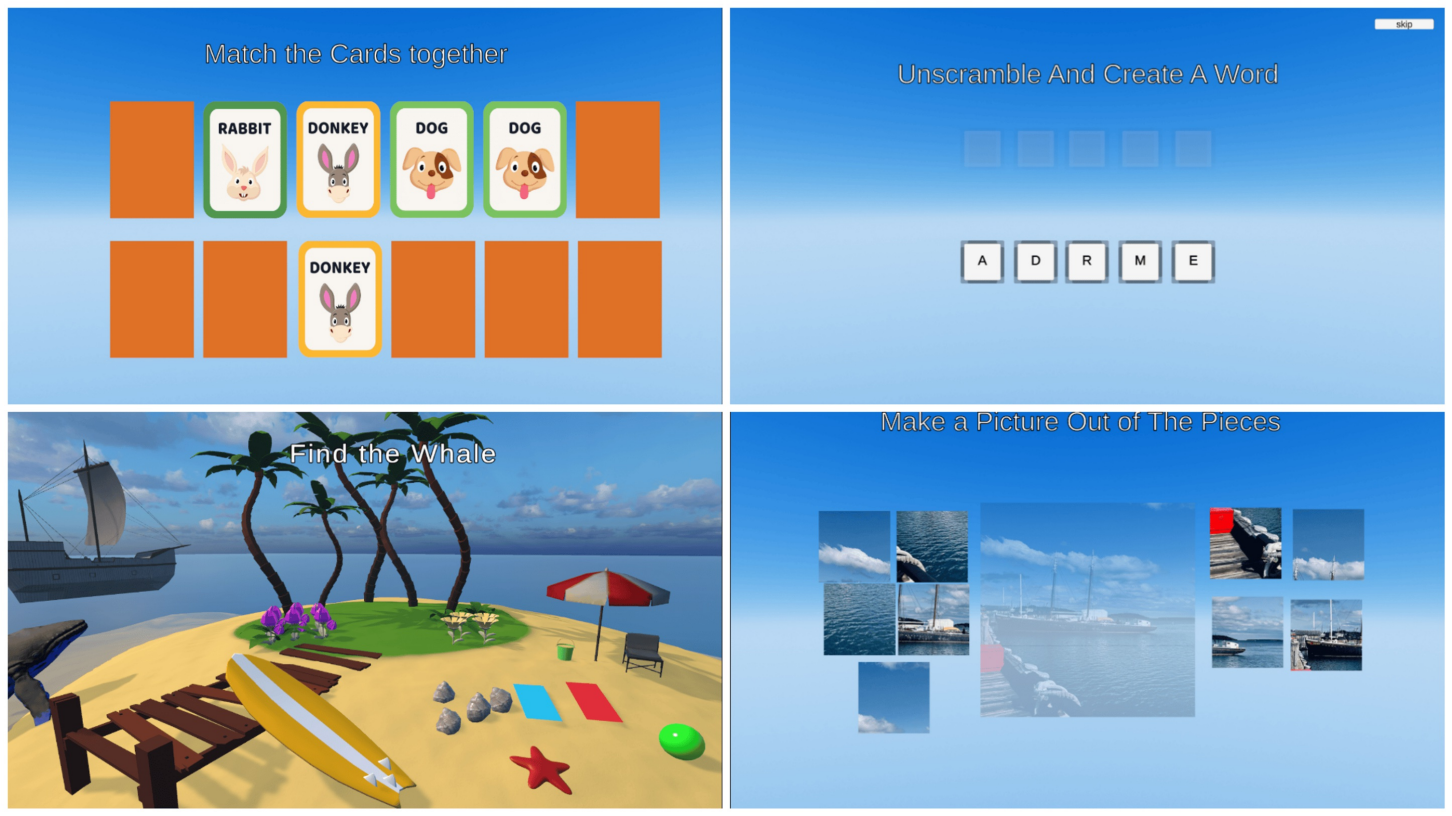
Cognitive stimulation activities Top left: matching cards. Top right: word scramble. Bottom right: jigsaw puzzle. Bottom left: find me game. Inage Credit: Authors' original creation.

Four different CS activities (Figure 3) were created in the Unity software and were designed by the main researcher (refer to Appendix A for the description of each CS activity). 

Matching of the Cards

Animals were chosen as the face of the cards because of their simplicity and the likelihood that participants have encountered these animals on a regular basis. Ten different animal cards were designed.

*Find Me Activity* 

Four distinct settings were created: park, beach, marina, and city center. Based on these scenes, questions were developed, such as asking a participant to find the ship in the marina. Each time the exergame was played, the scenes rotated. 

Jigsaw Puzzle

**Figure 4:**
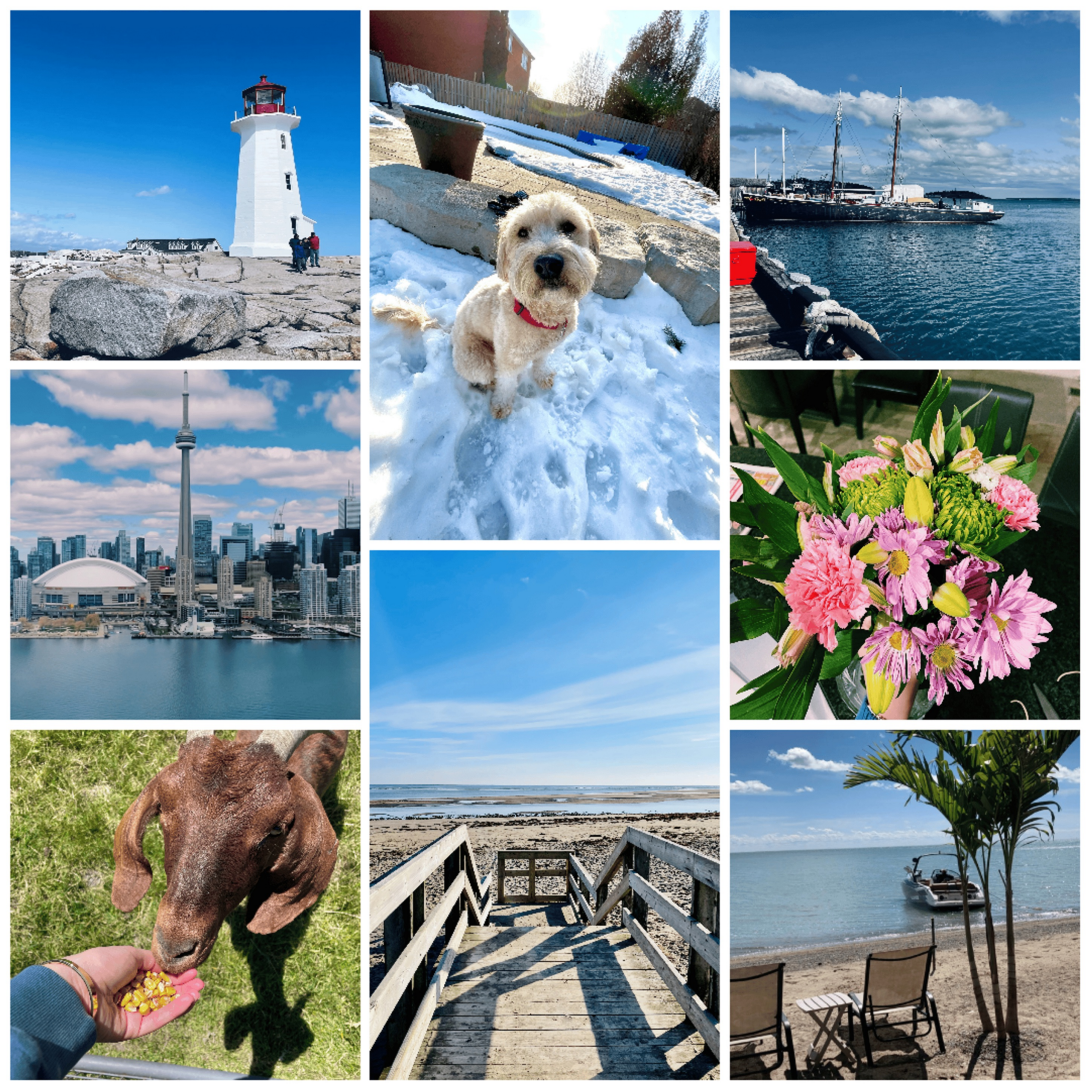
Jigsaw puzzle photo bank Image Credit: Author's original creation.

Nine distinct photos were compiled into a photo bank (Figure 4) and personally taken by the main researcher. Each photo was split into nine tiles, featuring images of locations or objects participants might have encountered in their lives. 

*Word Scramble* 

An English word bank of 20 words made up of four or five letters was prepared for the word scramble activity (Appendix B). Every exergame session used a different set of these words, and randomized. 

Assets were imported into Unity and instantiated into the scene for the user. Game mechanics [20] were coded to determine correct matches, decisions, or placements of puzzle pieces/letters. Feedback mechanisms [20] were implemented such as keeping matched cards face-up or locking puzzle pieces in place when correct. 

Metrics (accuracy, speed, and frequency of error) were integrated into the exergames tracking and reporting systems to measure the cognitive performance of the user. Targets were set to define correct actions, and an accuracy/error formula was applied. Task duration was tracked for speed, and results were displayed using Unity’s UI system, with a CSV file exported for review. 

Point system

A point system was introduced during Zumba exercises to enhance user motivation. This system was based on volumetric models created by the research team to assess movement accuracy. Real-time accuracy was evaluated by comparing participants' movements to the recorded models, focusing on the most and least distinct actions to avoid overwhelming the Kinect Azure. The Kinect Azure SDK tracked body movements through skeletal joints, translating them into game mechanics. Participants received a visual accuracy score from one to five stars based on their performance. 

Tech specifications 

**Figure 5:**
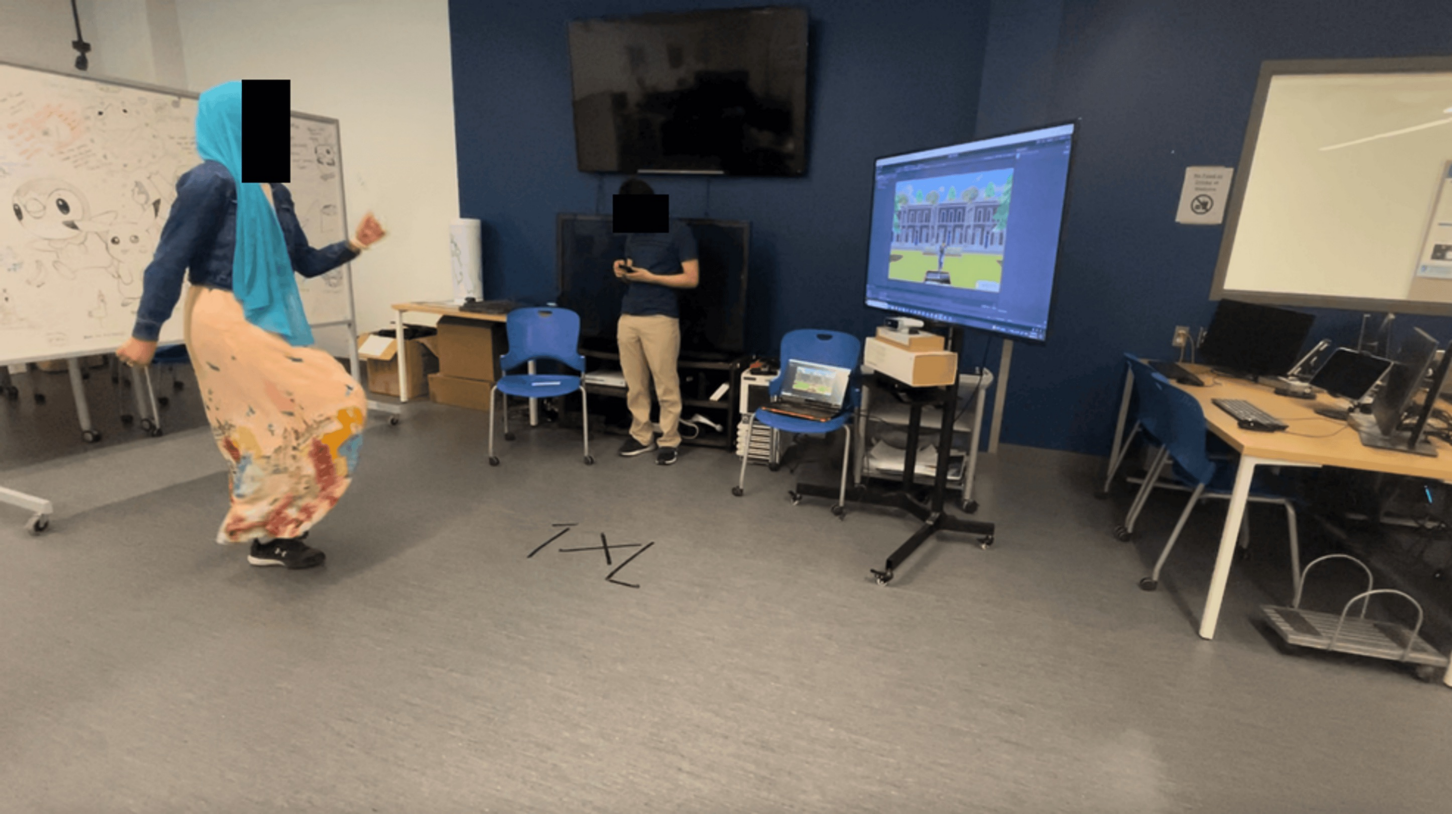
Exergame setup

The exergame requires a design set up of 2.00 m anteriorly, 3.00 m posteriorly, and 3.00 m laterally and an Azure Kinect placement of midlevel height of the participant, as shown in Figure 5. 

Refer to the following link for Azure Kinect’s technical requirements for the exergame [21]. Unity software and the body tracking SDK must be installed and capable of supporting the minimal SDK sensor in order for the computer to meet the specifications. Refer to the following links for more detailed information [21,22]. For a larger screen display, a television can be linked using an HDMI cable, and a computer mouse can be used to play on the computer CS activities. 

Pilot testing refinement

Three members of the research team pilot-tested with seven Ontario Tech University students and refined the exergame before the study began. The games' interactive features, movement speed, tracking system, and sizing were evaluated. Intervention dosage was also piloted to ensure the timing of the intervention was sufficient. Members of the research team ran through the exergame and tracked the average time to complete each CS activity. The team then doubled the average time to consider a clinical population with cognitive impairment. This was then piloted with two older adults with mild cognitive impairment from the community, where the intervention dosage was confirmed as appropriate. 

Data analysis

CS metrics were analyzed descriptively and presented in graphs. Qualitative semi-structured interviews were audio-recorded, transcribed verbatim, and analyzed in Dedoose using thematic analysis. An inductive coding approach was taken to gain a deeper understanding of the participants' perceptions, facilitators, and barriers to their experience. The emerging codes were organized into themes and sub-themes and represented using a code tree (Appendix C). The themes and sub-themes were then further generalized to represent the reported overarching themes from the study.

## Results

Background choices for participants 

**Figure 6:**
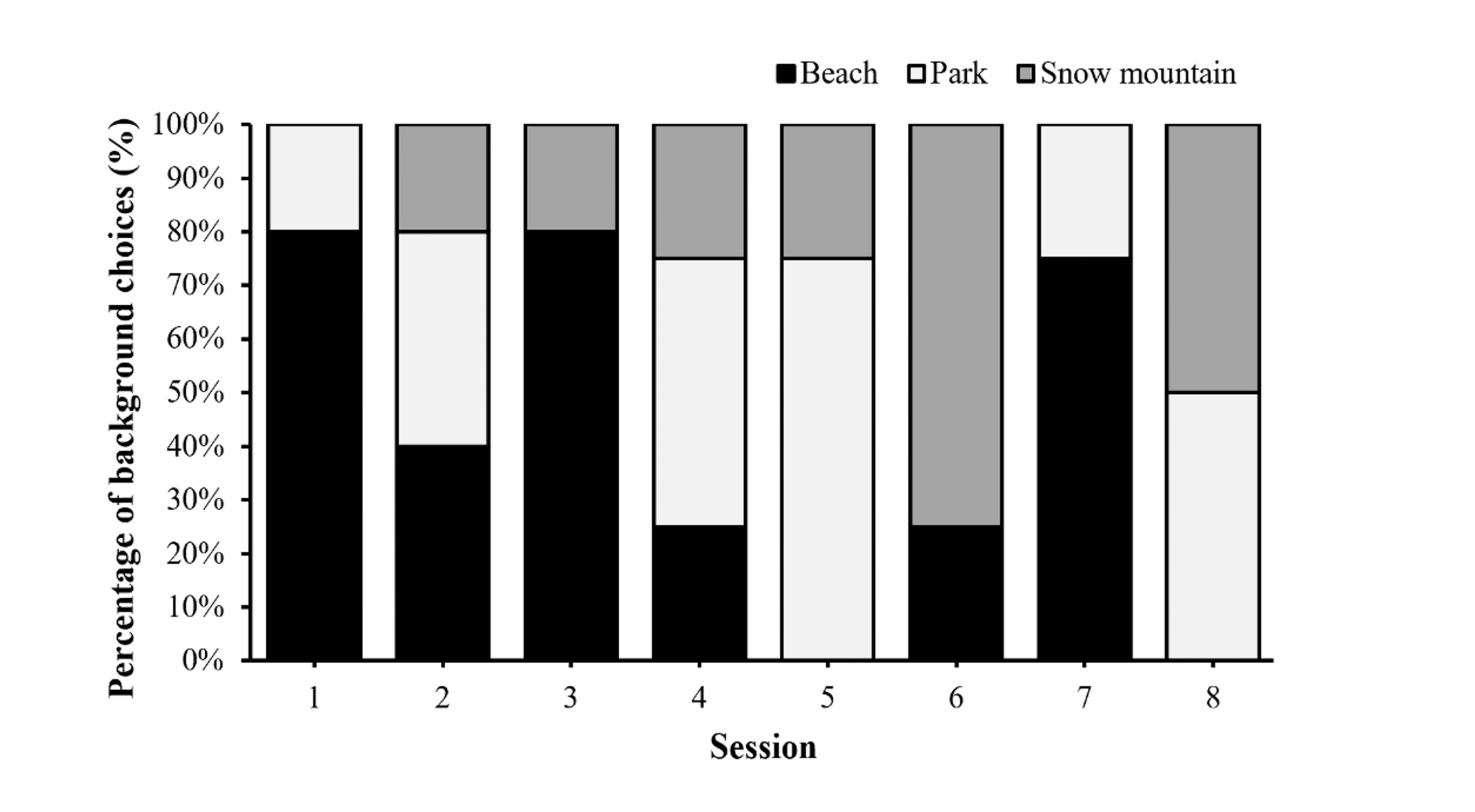
User background preferences Percentage distribution of background preferences across eight exergame sessions for five participants (n = 5). Each bar represents the count of selections for specific backgrounds (beach, park, snow mountain).

As shown in Figure 6, a beach background is the most favorable choice among participants, followed by a park background. The least common background choice selected was the snow mountain. The background selection was specific to the Zumba portion of the session. Participants were able to choose a different background for each session.

Cognitive stimulation metrics results 

Find Me Activity

**Figure 7:**
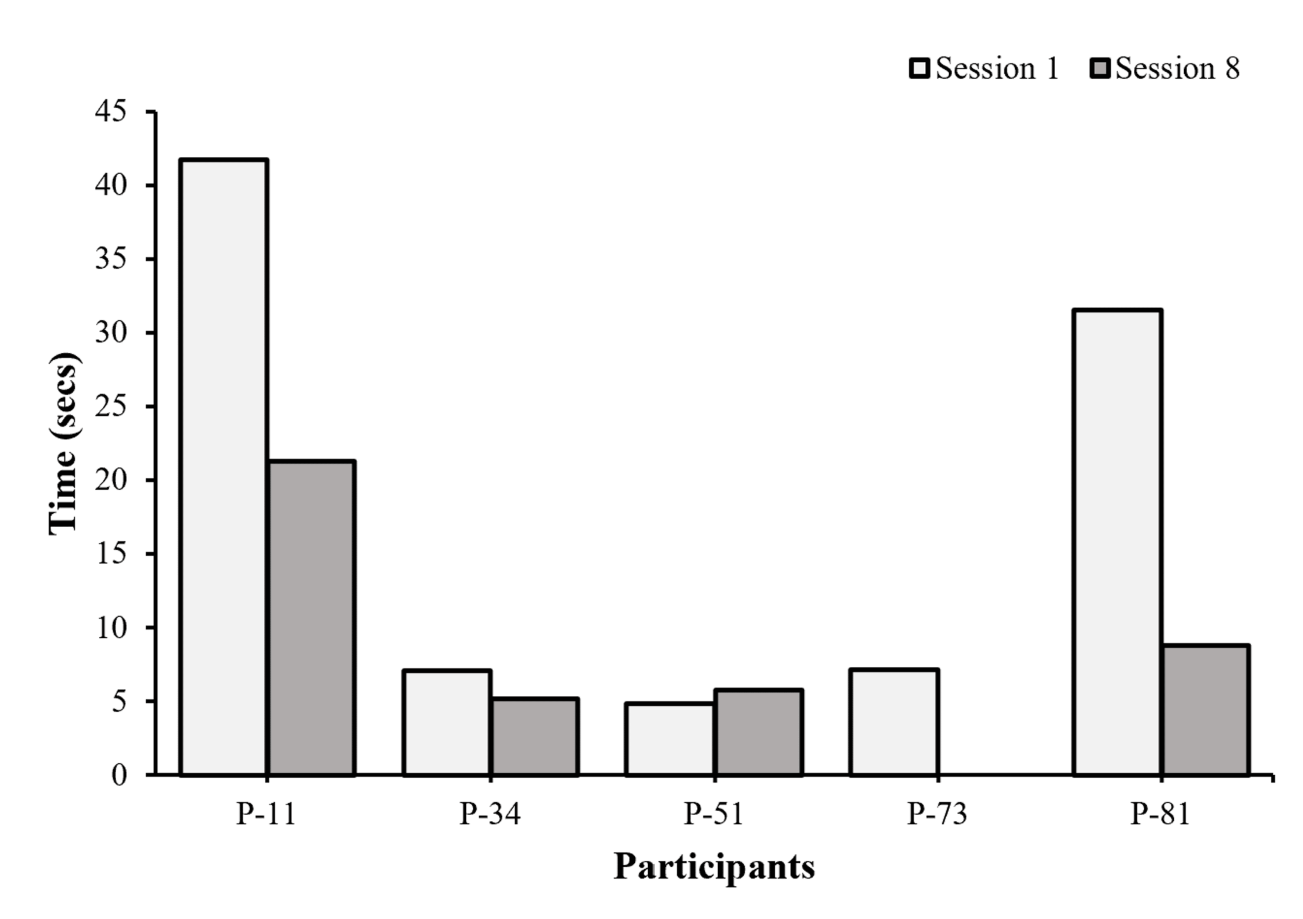
Find me activity time (speed) Each bar represents individual performance changes in speed pre- and post-intervention after eight sessions (n = 5).

Four participants saw an improvement in speed pre-/post-intervention (Figure 7). P-73 dropped out after session 3; therefore, her post-scores are missing. 

Matching of the Cards

**Figure 8:**
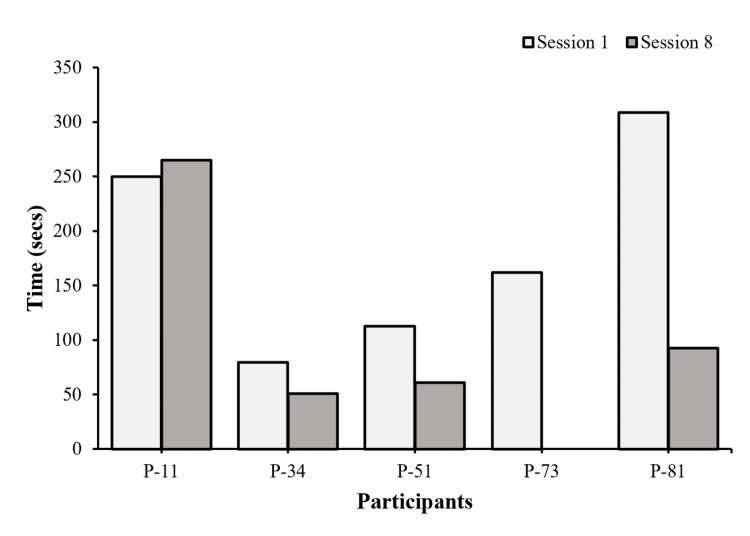
Matching Card Time (speed) Each bar represents individual performance changes in speed pre- and post-intervention after eight sessions (n=5).

**Figure 9:**
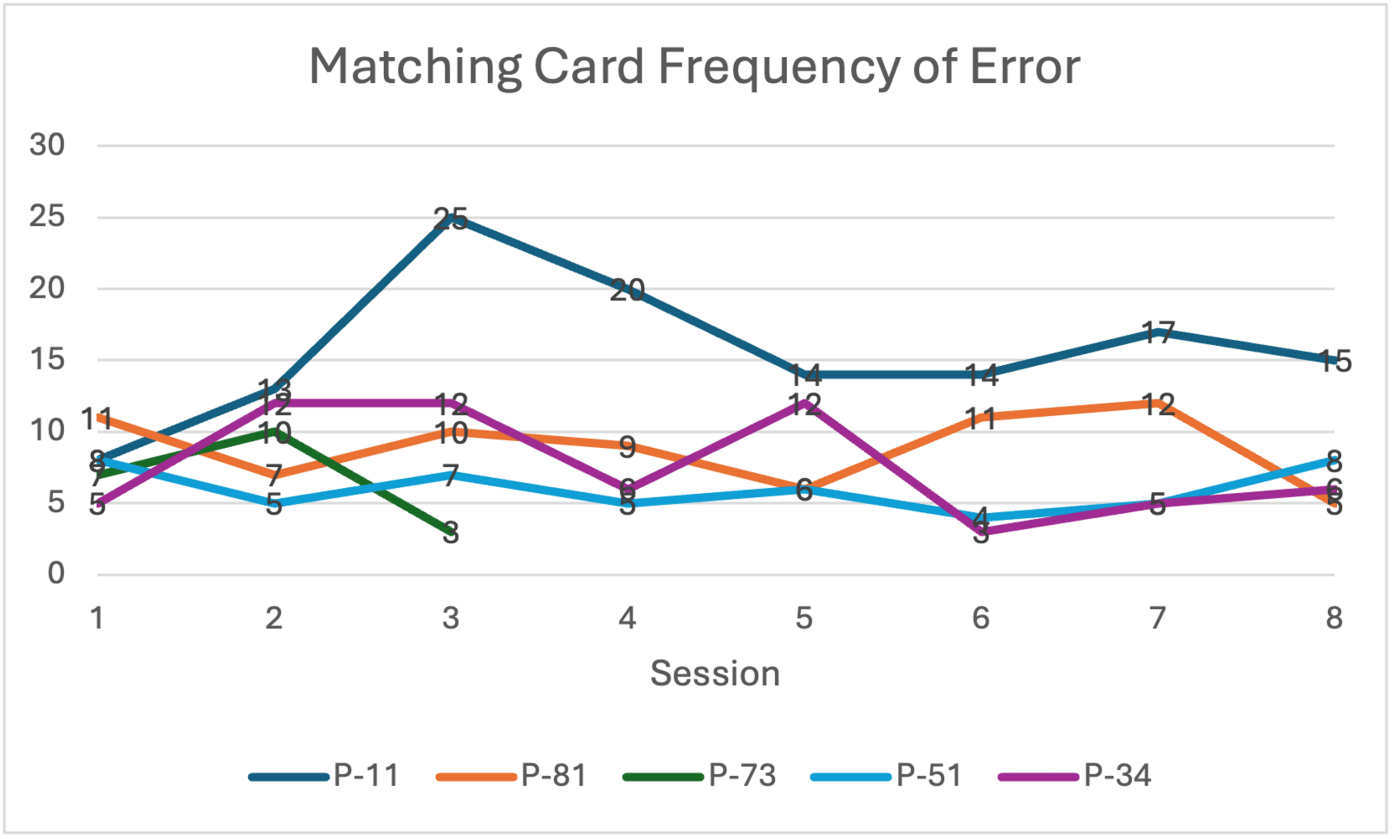
Matching card frequency of error Each line represents individual performance changes across eight sessions (n = 5).

Four participants saw an increase in speed and decrease in frequency of error (Figures 8, 9) pre-/post-intervention. P-73 dropped out after session 3; therefore, her post-scores are missing. 

Word Scramble

**Figure 10:**
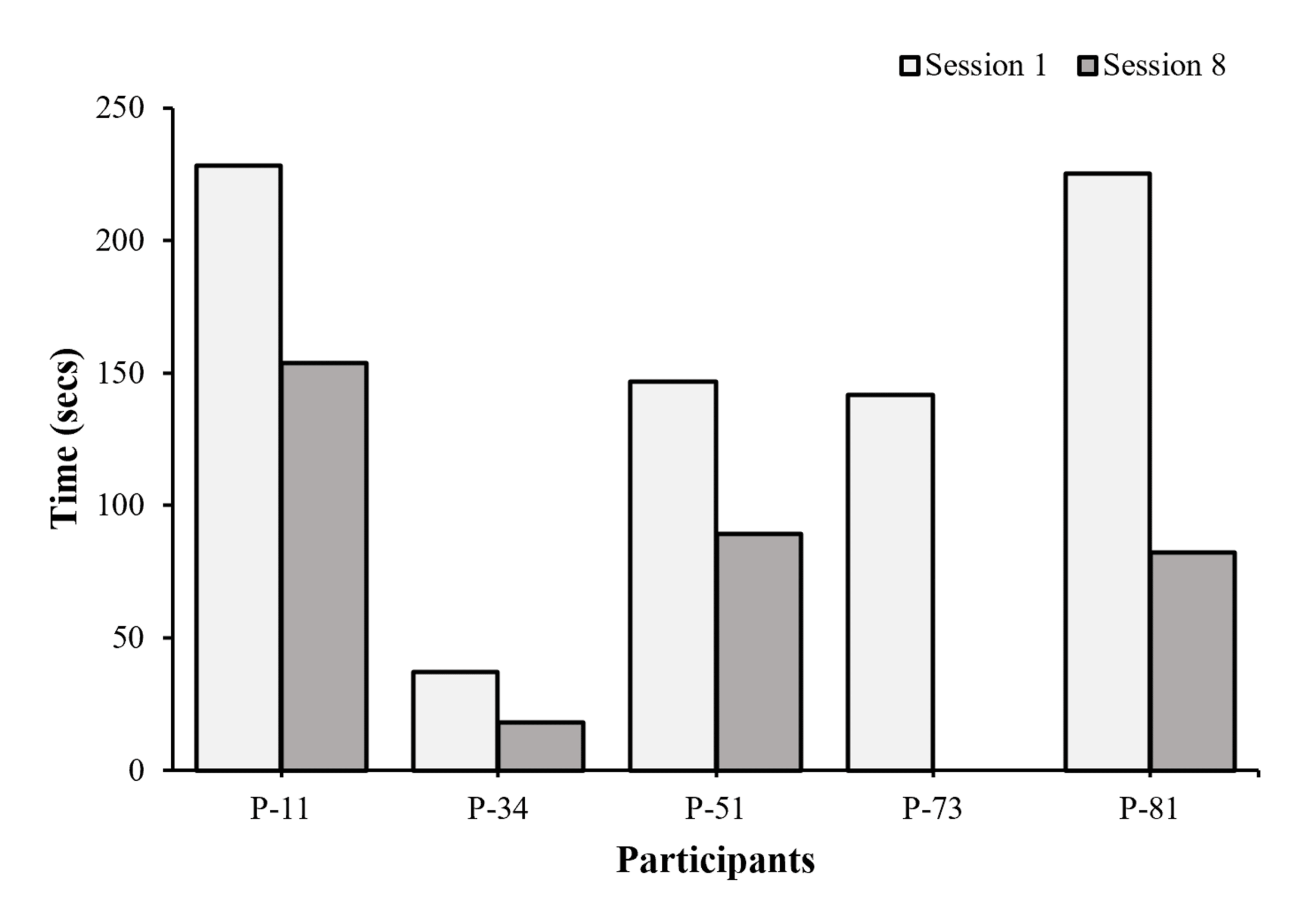
Word scramble time (speed) Each bar represents individual performance changes in speed pre- and post-intervention after eight sessions (n = 5).

Four participants saw an improvement in speed pre-/post-intervention (Figure 10). P-73 dropped out after session 3; therefore, her post-scores are missing. 

Jigsaw Puzzle

**Figure 11:**
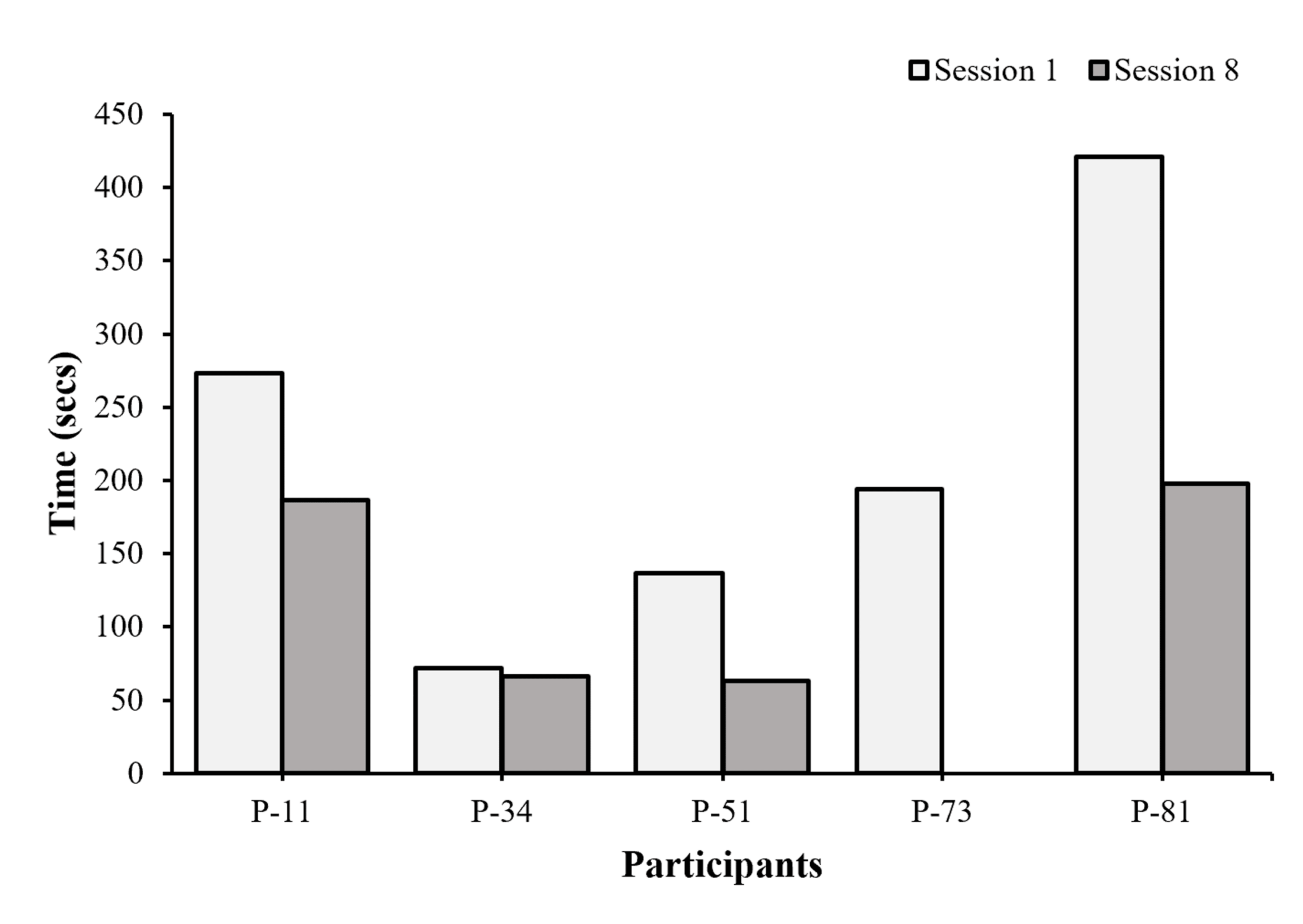
Jigsaw time (speed) Each bar represents individual performance changes in speed pre- and post-intervention after eight sessions (n = 5).

Four participants saw an improvement in speed pre-/post-intervention (Figure 11). P-73 dropped out after session 3; therefore, her post-scores are missing. 

Qualitative thematic analysis 

**Figure 12:**
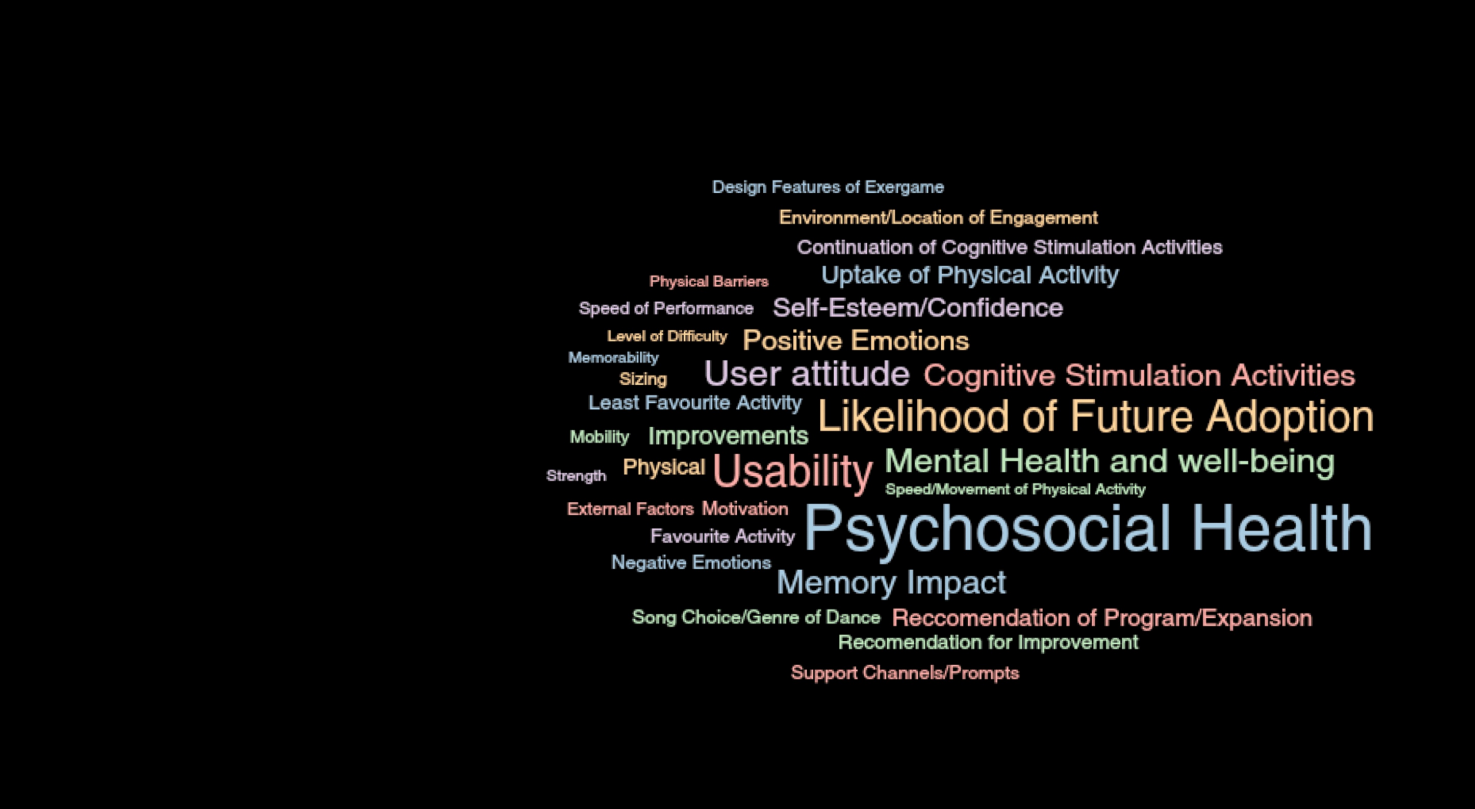
Thematic analysis The larger texts represent the larger themes the users commented on. Image Credit: Author's original creation.

Five participants completed semi-structured one-on-one interviews following the exergame, where six main themes were identified from the qualitative interviews: (1) usability, (2) physical health, (3) cognitive stimulation activities, (4) psychosocial health, 5) recommendations for improvement, and (6) likelihood of future adoption. The main overarching theme identified by the participants was psychosocial health (Figure 12).

Table 2 shows the thematic analysis results.

**Table 2:** Thematic analysis results: breakdown of overarching themes

Theme	Participants' perspective
Usability	Participants indicated that the exergame was a highly usable and friendly application. For example, one participant expressed that the exergame “combines mental alertness and physical activity. The combination is very good, I think” (P-73). A second participant quoted “It’s a pretty good program, I think. Makes yourself think and by participating I think that I am a little better for it” (P-11). Participants provided design feedback on the features chosen for the cognitive stimulation activities, such as the images helped with their cognitive recall. One participant noted “I liked it all because it made it stick in my head a little bit. Like this animal is here, and then oh I picked that one, but it was actually over there. So, it was good“ (P-51). Another participant quoted “The sizing was good, and the cards were good like the images that I picked to get the puzzle together. The mouse was easy to move, to make a puzzle or click on the animals for the cards” (P-51). Further probing the design features of the exergame, participants felt that it was effective at improving their cognitive skills such as attention to detail and memory recall/retention, translating to positive psychosocial elements in individuals’ daily lives as evident in their feedback. One participant noted “it was fun trying to figure it out. I had to pay attention to detail, and I seemed to do a bit better” (P-73), while another participant voiced that their confidence grew while playing the jigsaw or matching card games “Now I am thinking that I must remember where that was or goes. Before it was just random, so now I am more confident” (P-81).
Physical health	Participants also noted physical improvements within themselves after eight sessions of exergaming. For example, one participant explained “at the beginning of the program my muscles were getting stronger because of the exercises from the combination of the exergame and the exercises I was asked to do” (P-73). While other participants added “I feel like my torso has gotten stronger. I am more confident moving side to side and rotating” (P-81), and “I don’t have as much pain anymore in my back” (P-34). These results highlight exergaming’s potential in providing an environment for individuals to engage in physical activity as well as increase the uptake within their daily lives. Exergaming participation also highly impacted the confidence and self-esteem of participants and provided them with a sense of well-being “Because it's enjoyable. I feel success, and you (RA) make it very enjoyable. You feel like you are accomplishing something when you do it” (P-81) and “I didn’t feel useless. I felt like I was accomplishing something” (P-81). These findings demonstrated that exergaming has the potential to impact multiple pillars of health, such as cognitive, physical, and psychosocial.
Cognitive stimulation activities	Participants were asked which activities were their most favorite and responded by noting their enjoyment in playing matching of the cards and “find me” game. One participant voiced enjoying it more because they were able to locate items easily “Yes, I got better. I was thinking more about it and was trying to remember “oh yes the cat was over there” (P-81) or because of the repetition of the tasks such as the matching of the cards, “Because every time I did it, I thought I did better” (P-34). On the contrary, some participants found this activity challenging. For example, “my concentration to remember where they were, and I tried to remember but after I would answer the second card and get it wrong, then I lost my concentration to it" (P-11). This could be related to the short-term memory struggles that some participants may have due to their cognitive impairment.
Psychosocial health	The favorability of activities indicated by the participants had transcribed into positive psychosocial elements. For example, one participant noted “because I can feel some success... because the more cards I got right, the better I felt” (P-81). When asked to reflect upon their memory confidence or recall, a participant explained “I think it has improved, yes. I can think of things that I have done here and tell my (XXX) when I go out what I did. (XXX) always asks me, and then I can tell (XXX) “Oh I got five stars today” and I am able to remember” (P-51). These quotes highlight the potential impact of exergame to instill confidence within participants as they engage in either physical activity or cognitive tasks, and these positive feelings can translate into better psychosocial health outcomes, such as positive moods, quality of life, and an overall sense of well-being.
Recommendation for improvement	Although there were suggestions to increase the size of the exergame avatar and to slow some of the physical activity movements, all participants expressed a desire to recommend it to other individuals with similar needs. For example, one participant explained “I am glad I got to participate, and I hope through this it will maybe help me even more or someone else” (P-81), while other participants noted “If you are going to do this again, I would like to be part of it again, and for those who are thinking of signing up, its enjoyable and it’s worth it” (P-34) and “Because it’s helping me, and I am sure it would help others or someone else” (P-11) further demonstrate that exergaming programs have the potential to help.
Likelihood of future adoption	Multiple participants also expressed that the combination of physical activity and cognitive challenges was motivating and enjoyable. One participant described “Because it gets you up, and out of bed. It gives you something to do and get yourself ready. You don’t wear the same clothes every day, you can wear something different and just doing the exercises is really more fun than anything that I have done. While another participant highlighted “I have really enjoyed both the exercises and the memory games” (P-51). And finally, other participants highlighted that the exergame had improvements on their cognition and well-being: “Mentally, I am more aware, and I talk more. as I am a very quiet person. Now I reach out to people, and I spend time with my (XXX), which makes me happy” (P-34). These quotes demonstrate the potential impact of exergaming on individuals’ psychosocial health and how the uptake of physical activity increases when enjoyable environments are created or established. Although, there were suggestions to increase the size of the exergame avatar and to slow some of the physical activity movements, all participants expressed a desire to recommend it to other individuals with similar needs. For example, one participant explained “I am glad I got to participate, and I hope through this it will maybe help me even more or someone else” (P-81), while other participants noted “If you are going to do this again, I would like to be part of it again, and for those who are thinking of signing up, its enjoyable and it’s worth it” (P-34) and “Because it’s helping me, and I am sure it would help others or someone else” (P-11) further demonstrate that exergaming programs have the potential to help.

Table 3 presents the summary of the lessons learned from participants' feedback. 

**Table 3:** Summary of the lessons learned CS: cognitive stimulation, PA: physical activity.

Most enjoyable
Combination of CS and PA was feasible
Images of each of the CS activities
Beach background was chosen the most
Participants expressed they liked animals for the design, stating it was memorable, and they felt confident and successful as they continued to play
The music and length of the program
Most favorite activities
Find me game (liked picking out objects within a scene)
Matching of the cards (liked finding the pairs and animals)
Jigsaw puzzle
Zumba
Least favorite activities
Word scramble: participants found it harder to sort out the letters to form the word (challenges with concentration)
Jigsaw puzzle: one participant found it hard to concentrate (short-term memory implications)
Future exergame considerations
Increase the avatar size
Slow (reduce the speed)/have more repetitions of the Zumba movements

## Discussion

This pilot cohort feasibility study consisted of a four-week exergame program with five individuals aimed at improving cognitive function through PA and CS while interacting with the exergame. In the literature, exergames have gained attention for their potential to improve the uptake of PA and improve overall physical fitness, cognition, and mobility [4,5,23]. The gamified nature has the potential to increase motivation and engagement [7], which is crucial for sustained participation and uptake. 

The qualitative themes identified within this study revealed that the exergame design was beneficial and potentially helped participants cognitively, physically, and psychosocially. Previous studies by Mura et al. [24] and Karssemeijer et al. [25] found evidence of these findings as well. By incorporating playful elements like CS activities and Zumba into a game-like setting, participants can improve both cognitive and physical health [24,25]. Similar findings were noted by van Santen et al. who conducted an RCT study and secondarily explored the physical, cognitive, and social functioning of individuals with dementia and their care experiences [26]. Their findings found positive effects on competence and enjoyment [26]. This aligns with our study findings, where participants felt working on a puzzle or task fostered a sense of autonomy and achievement. 

Current literature shows a lack of feasible, personalized programs for individuals with cognitive decline to engage in both PA and CS activities. Thematic analysis identified key lessons such as preferred and disliked activities and design elements, and the reasons behind them. Félix et al. highlighted the importance of design considerations for exergames, including PA type, dosage, engagement methods, and psychosocial benefits [7]. This study aimed to provide insights for future researchers to design feasible, personalized programs for individuals with cognitive impairment. Woods et al. found evidence of this as well, indicating that the intervention dosage and severity of diagnoses may influence outcomes and the mode of delivery (virtual or remote) [27]. Overall, the literature lacks detailed reporting on the benefits of exergaming interventions. This study aimed to be transparent about the design, development, and intervention details, as well as lessons learned, to guide future researchers in conducting similar studies. 

Limitations

This pilot study had a small sample size (n = 5), as such results should be interpreted as preliminary. One participant dropped out after the third session, due to personal reasons, unrelated to the study intervention; however, this participant still completed the qualitative interview. A design limitation was the potential for “the practice effect," or a learning curve [28]. Although this study tried to minimize these biases through randomizing sequences, word banks, or picture rotations, they should still be considered. Future studies should incorporate the lessons learned and implement co-design workshops to gather end-user feedback for better engagement and personalized experiences. Additionally, accessibility considerations, such as offering language options (French or Chinese) in the word scramble, should be reviewed to create a more culturally diverse design. 

## Conclusions

This pilot mixed methods study with five study participants was found feasible and suggests that exergaming may hold potential benefits for persons with dementia and LLD by promoting PA and CS engagement. Future studies should apply and build on the lessons learned and qualitative feedback gathered by adopting a co-design approach in a larger sample size to create relevant and feasible interventions that can be effectively implemented into clinical practice for these populations. 

## Figures and Tables

**Figure 13:**
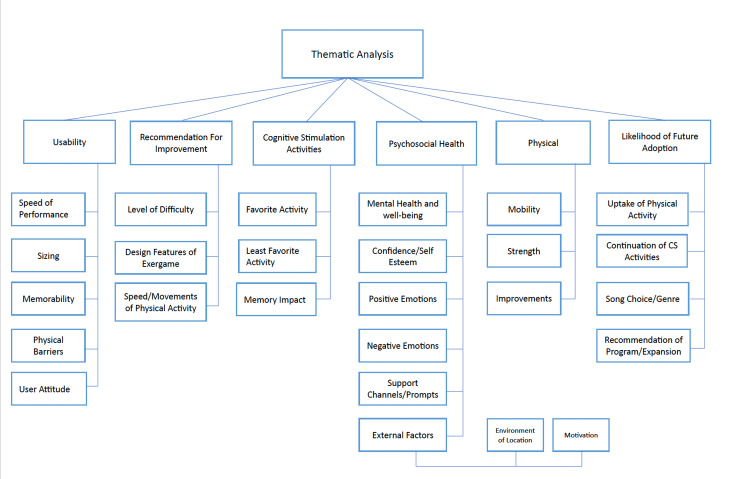
Coding tree

## Appendices

Appendix A

**Table 4:** Description of cognitive stimulation activities

Matching card game
Use the mouse and click on two cards to flip them over. If the two cards match, go again. If they do not match, pick two new cards to flip until you find a pair. Go until all the pairs are found.
Find me game
A question will appear on the screen. Participants must find the object within the scene and click on it using the mouse.
Example: Find the seashells.
Participants will be asked four questions.
Jigsaw puzzle
Participants will be shown a reference photo, and there will be square pieces scattered on the outside border.
Participants will click and drag the correct piece onto the spot it belongs. If a piece does not belong, it will not lock in place.
Once the puzzle is complete, the green box in the corner will light up green.
Word scramble
Participants will be given tiles with letters on them and will have to spell out the correct word. They will click and drag the correct tiles into the spaces outlined on the screen.
Participants will complete 4-5 words, four or five letters long.

Appendix B

**Table 5:** Word scramble word bank

Word	Letters
Fork	ROFK
Bell	LLEB
Grow	WOGR
Fish	SHIF
Blow	OWBL
Burn	RNUB
Hold	LDOH
Bowl	WLBO
Wash	SHWA
Menu	NUEM
Berry	RRYBE
Water	RETWA
Heart	RTEAH
Beach	CHBEA
Flash	SHFLA
Birds	DSIRB
House	SEOUH
Cabin	BINCA
Dance	CENDA

Appendix C
